# The immune checkpoint molecule, VTCN1/B7-H4, guides differentiation and suppresses proinflammatory responses and MHC class I expression in an embryonic stem cell-derived model of human trophoblast

**DOI:** 10.3389/fendo.2023.1069395

**Published:** 2023-03-16

**Authors:** Jie Zhou, Yuchen Tian, Ying Qu, Madyson Williams, Ye Yuan, Rowan M. Karvas, Megan A. Sheridan, Laura C. Schulz, Toshihiko Ezashi, Michael R. Roberts, Danny J. Schust

**Affiliations:** ^1^ Department of Obstetrics, Gynecology, and Women’s Health, University of Missouri, Columbia, MO, United States; ^2^ Bond Life Sciences Center, University of Missouri, Columbia, MO, United States; ^3^ Department of Obstetrics and Gynecology, Duke University Medical Center, Durham, NC, United States; ^4^ Research Department, Colorado Center for Reproductive Medicine, Lone Tree, CO, United States; ^5^ Department of Developmental Biology, Washington University School of Medicine, St. Louis, MO, United States; ^6^ Department of Biochemistry, University of Missouri, Columbia, MO, United States; ^7^ Division of Animal Sciences, University of Missouri, Columbia, MO, United States

**Keywords:** embryonic stem cells, placental development, B7-H4/VTCN1, classical major histocompatibility complex class I molecules (MHC-I), natural killer cells, HLA-G, virus, anti-viral responses

## Abstract

The placenta acts as a protective barrier to pathogens and other harmful substances present in the maternal circulation throughout pregnancy. Disruption of placental development can lead to complications of pregnancy such as preeclampsia, intrauterine growth retardation and preterm birth. In previous work, we have shown that expression of the immune checkpoint regulator, B7-H4/VTCN1, is increased upon differentiation of human embryonic stem cells (hESC) to an *in vitro* model of primitive trophoblast (TB), that VTCN1/B7-H4 is expressed in first trimester but not term human placenta and that primitive trophoblast may be uniquely susceptible to certain pathogens. Here we report on the role of *VTCN1* in trophoblast lineage development and anti-viral responses and the effects of changes in these processes on major histocompatibility complex (MHC) class I expression and peripheral NK cell phenotypes.

## Introduction

Study of the immunology of the human maternal-fetal interface has been hampered by several inherent characteristics of human placentation. First, the immunologic events at the maternal-fetal interface are dynamic, with features of pro-inflammatory responses at the initiation of pregnancy and near the time of delivery, and more pro-tolerogenic traits during much of the interval in-between (1). Immune characteristics also differ by location, as the placenta produces large amounts of immunomodulatory hormones and cytokines and presents high levels of allogeneic antigen load that decrease with distance from this source, i.e., antigenic load and immunomodulatory pregnancy hormone levels are generally lower in the maternal periphery than within the pregnant uterus (2–4). The human placenta is notably invasive, with fetal-derived, allogeneic placental trophoblast (TB) cells invading deeply into the maternal decidua and into the inner layers of the uterine myometrium. These invasive extravillous trophoblast cells (EVT) also remodel the decidual spiral arteries, where they are called endovascular trophoblast (endoTB) cells and are in direct contact with maternal peripheral blood, including its immune components. This level of invasion and subsequent interactions of 1) EVT with decidual immune cells, and 2) endoTB and the syncytiotrophoblast (STB) of the villous placenta with immune cells in the peripheral blood, is unusual among non-primate eutherian mammals, making small animal modeling of the human placenta problematic. Here, to address the immune events surrounding human implantation, we utilize two of a very limited number of *in vitro* models of preclinical human pregnancy: a human embryonic stem cell (hESC)-derived model of primitive TB (BAP cells) and, for validation, human blastocyst extended culture.

Two main models have been proposed to explain the immunology of human placentation (5–7). The first compared the implanting fetus to an allotransplant, since both represented an allogeneic mismatch between an immunologically competent host (mother/recipient) and foreign tissue (implanting fetus/transplanted organ) (7, 8). The second compared the implanting fetus to an invasive tumor (9), since both require robust cellular proliferation and “tumor” growth, rapid and extensive neovascularization, invasive capabilities, and local immunomodulation to avert rejection. One could even compare the long-term presence of fetal cells in the maternal periphery, a process called fetal-maternal microchimerism, to tumor metastasis. Although, for several reasons, we favor the tumor paradigm, neither fully reflects the nuanced immune changes that occur in the pregnant woman, particularly at the maternal-fetal interface. In short, the implanting fetus is neither an allogeneic transplant nor an invasive tumor, and its immunology is complex. For example, while a tumor can induce a local immunotolerant microenvironment, the immune cells at the site of the implanting fetus actively prepare the decidua for its implantation. Still, knowledge gained from transplant and cancer immunology can help to guide experimental direction and stimulate hypotheses during the study of pregnancy immunology. Accordingly, molecular regulators active in cancer and transplant immunology, may potentially play a role in immunomodulation at the maternal-fetal interface.

In RNA-seq analyses comparing BAP cells to term TB syncytialized in culture, we identified several genes that were differentially expressed at high levels in primitive TB (10) and verified that expression of the corresponding proteins was highest in early pregnancy but waned over gestation in primary human placental tissues (11). One of these genes was V-set domain-containing T cell activation inhibitor 1 (*VTCN1*), whose gene product is a cell surface-expressed protein belonging to the B7 costimulatory family, most often called B7-H4 in the immunology literature. *VTCN1* is typically expressed on the surface of antigen presenting cells, and although its precise receptor on the surface of T cells has not yet been determined, its role is likely as an immune checkpoint regulator. In most studies, the neo expression of *VTCN1*/B7-H4 on cancer cells is associated with cancer progression and poorer prognosis (12–16). Its presence in our model of peri-implantation human TB prompted us to hypothesize that it promotes maternal immune tolerance of trophoblast and/or trophoblast response to pathogens. Secondly, given the frequent comparisons of the implanting fetus to an invasive tumor (17, 18), we further hypothesized that *VTCN1*/B7-H4 could play a role in trophoblast invasion. Here, the BAP model of peri-implantation TB is used to test both hypotheses.

## Materials and methods

### Human ESC culture and differentiation

Human ESCs (H1, WA01) were cultured in Matrigel (BD Bioscience)-coated, six-well tissue culture plates (Thermo Scientific) under an atmosphere of 5% (vol/vol) CO2/air at 37°C in mTeSR1 medium (STEMCELL Technologies). The culture medium was changed daily and the cells were passaged every 5-6 days. The method for TB differentiation has been described previously (19). Briefly, on the day after passaging onto Matrigel-coated dishes at 1.2 × 10^4^ cells/cm^2^, the culture medium was changed to DMEM/F-12 medium (Thermo Scientific) with knock-out serum replacement (KOSR, Invitrogen) that had been conditioned by mouse embryonic fibroblasts (MEF) and supplemented with FGF2 (4 ng/mL). After 24 h, the conditioned medium was replaced with daily changes of nonconditioned DME/F12/KOSR medium lacking FGF2, but containing BMP4 (10 ng/mL), A83-01 (1 μM), and PD173074 (0.1 μM) (BAP treatment) for up to 8 days. Control cultures were maintained in conditioned medium containing 4 ng/mL FGF2.

### PBMC isolation and coculture with TB cells

Human subjects were recruited for participation under an IRB approved protocol (University of Missouri, Columbia, Office of Human Research Protection Program, Medical IRB Committee-1 #2017804). Each subject provided written informed consent prior to enrollment. Peripheral blood was collected from 13 healthy women in the first trimester of pregnancy (6-12 wks estimated gestational age). The PBMC fraction was isolated from peripheral blood by using Ficoll-Paque Premium (GE Healthcare, Pittsburgh, PA) and centrifugation at 400×g for 30 min at 20°C; cells were cryopreserved until use.

PBMCs were thawed in pre-warmed (37°C) FBS. PBMC viability was over 80% as judged by Trypan blue (Sigma-Aldrich, St. Louis, MO) exclusion. After washing, PBMCs were resuspended at 5 X 10^5^ cells/mL in nonconditioned DME/F12/KOSR medium containing IL-2 (100U/ml) and co-cultured with BAP treated TB cells for 72 h (BAP d5-d8) at 37°C.

### Flow cytometry

BAP-primed hESC cells were dissociated with ACCUTASE™ (STEMCELL™ TECHNOLOGIES) at 37°C for 10 min. Cells were washed in culture medium and passed through a 40-µm cell strainer to remove the larger STB population (Fisher Scientific). Cells were placed in 3% (wt/vol) BSA in PBS for 30 min to minimize non-specific interactions with antibody and stained with APC-conjugated anti-human HLA-G monoclonal mouse antibody (BioLegend, 335910). After being washed twice with PBS, cells were fixed in 4% (vol/vol) paraformaldehyde (PFA) in PBS. APC-conjugated mouse IgG2a Isotype control (BioLegend, 40222) antibody was employed to account for nonspecific staining. Cells were analyzed in a BD Accuri™ flow cytometer (BD Biosciences, San Jose, CA) with subsequent FlowJo software (Tree Star, Ashland, OR). Suspended cells from the co-culture were harvested for flow cytometry 72 h after co-culture establishment. Cells were washed and stained with the following antibodies: PE/Dazzle™ 594 conjugated anti-CD3 Ab (BioLegend 300336), APC-conjugated anti-CD56 Ab (BioLegend 362504) and phycoerythrin (PE)-conjugated anti-CD16 Ab (BioLegend 302008) for 30 min at 4°C. After washing twice with PBS, cells were fixed in the *Foxp3*/transcription factor staining buffer (eBioscience) according to manufacturer’s instructions. Matched fluorescence-labeled isotype antibodies were used as a negative control to account for nonspecific staining. Immunostained cells were analyzed by flow cytometry as described above.

### RNA interference and crystal violet assay

For small interfering RNA (siRNA)-mediated knockdown of *VTCN1*, differentiated hESCs were transfected with either a *VTCN1* human siRNA oligo duplex (Origene, SR312516, 10nM) or a control, Trilencer-27 Universal scrambled negative control siRNA duplex (Origene, SR30004, 10 nM) on BAP treatment d 3 by using Lipofectamine 2000 (Invitrogen). After 6 h, the medium was replaced with BAP medium. Total RNA was collected 24 h, 48 h and 72 h after transfection. Protein was collected 72h after transfection. At BAP treatment d 6, i.e., 72 h after the BAP 3 days, cells colonies were stained with crystal violet solution [0.2% wt/vol crystal violet in an aqueous solution containing 20% (vol/vol) ethanol and 10% (vol/vol) formaldehyde] for 15 min. After washing with water, images of individual cell colonies were captured with a Leica M205 stereoscope.

### *VTCN1* ^-/-^ hESC line

**Figure 1:**
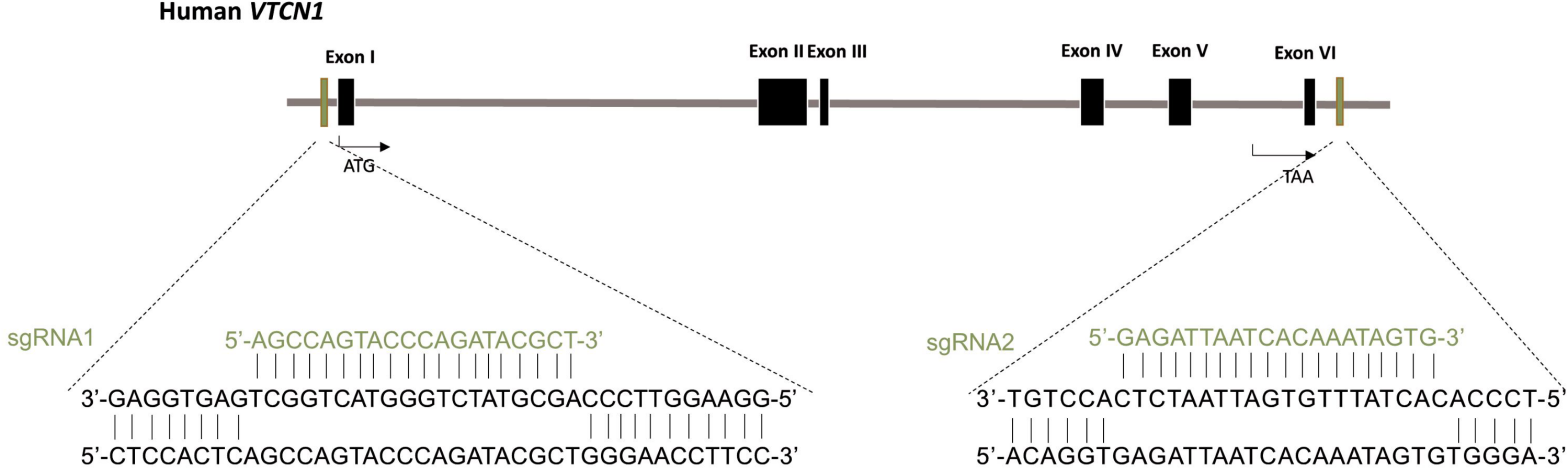
Generation of VTCN1-/- hESC using CRISPR/Cas9 genome editing. Schematic of the base pairing between guide RNAs (sgRNA1 for upstream and sgRNA2 for downstream) for VTCN1 and their targeting loci within VTCN1.

*VTCN1* has five isoforms and our study showed CRISPR gRNA/Cas9 at a single site mutation was not sufficient to knockout the isoform products. To overcome it, two gRNAs were used to delete the whole coding sequence. The *VTCN1* ^-/-^ cell line was generated from the hESC by CRISPR nuclease-induced, targeted, double-strand breakage at the Genome Engineering and iPSC Center (GEiC), Washington University in St. Louis. Briefly, gRNAs were designed to target an exon common to all transcripts, where an indel can lead to nonsense mediated decay of the messenger RNAs. The parental H1 cells were nucleofected with the CRISPR gRNA/Cas9 ribonucleoprotein complex, and single cell H1 clones were screened for indels or biallelic deletions in the target gene (*VTCN1*). The whole coding sequence of *VTCN1* was targeted with two guide RNAs: sgRNA1: 5’-AGCCAGTACCCAGATACGCT-3’ and sgRNA2: 5’-GAGATTAATCACAAATAGTG-3’ (**Figure 1**). Genomic PCR determined biallelic deletion of *VTCN1* gene.

### RNA extraction and quantitative real-time PCR

RNA was extracted in STAT60 containing 1-bromo-3-chloro-propane (Sigma-Aldrich), and DNA removed by using the TURBO-DNA-free kit (Ambion Inc.). A total of 650ng of each RNA sample was included for complementary DNA synthesis by using the iScript™ cDNA Synthesis kit (BioRad). Primers were designed based on published sources and synthesized by Integrated DNA Technologies; primer sequences are summarized in **Table 1**. Quantitative RT-PCR (qRT-PCR) was performed with a SYBR Green qPCR Master Mix (MedChemExpress) on the BioRad CFX Connect Real-Time PCR Detection System. Thermal cycling conditions were as follows: 95°C for 5 min, followed by 45 cycles of: denaturation at 95 °C for 10 sec, annealing at 56 °C for 10 sec and extension at 72 °C for 10 sec. PCR normalization of data to that of the endogenous control (GAPDH) and fold-change values calculated by the 2^−ΔΔCT^ procedure have been described elsewhere (20). The final PCR results were expressed as relative expression compared to individual control sample in each assay. Data followed over time were subjected to two-way ANOVA, followed by the Bonferroni test for pairwise comparisons. Values of p<0.05 were considered to support the conclusion that differences were statistically significant.

**Table 1:** Primers used for Qpcr.

Gene	Primer sequence
*VTCN1*	5′GGG GAG GAT GGA ATC CTG AG
	5′CTC CGA CAG CTC ATC TTT GC
*HLA-A*	5′AAA AGG AGG GAG TTA CAC TCA GG
	5′GCT GTG AGG GAC ACA TCA GAG
*HLA-B*	5′GAC GGC AAG GAT TAC ATC GCC CTG AA
	5′CAC GGG CCG CCT CCC ACT
*HLA-C*	5′GGA GAC ACA GAA GTA CAA GCG
	5′CGT CGT AGG CGT ACT GGT CAT A
*HLA-E*	5′CCT ACG ACG GCA AGG A
	5′CCC TTC TCC AGG TAT TTG TG
*HLA-G*	5′CTC TCA GGC TGC AAT GTG AA
	5′CAT GAG GAA GAG GGT CAT GG
*CGB*	5′GTC AAC ACC ACCACC ATG TGT GC
	5′GGT AGT TGC ACA CCA CCT GA
*ISG15*	5'GAG AGG CAG CGA ACT CAT CT
	5'CTT CAG CTC TGA CAC CGA CA
*OAS1*	5′GCG CCC CAC CAA GCT CAA GA
	5′GCT CCC TCG CTC CCA AGC AT
*MX1*	5′TGG CAT AAC CAG AGT GGC TG
	5′CAC CAC CAG GCT GAT TGT CT
*GAPDH*	5′ CTG GGG CTG GCA TTG CCC TC
	5′ GGC AGG GAC TCC CCA GCA GT

### RNA-seq analyses

RNA was obtained from *VTCN1* knock down or negative control siRNA transfected cells 24 h, 48 h and 72 h after transfection. The experiment was performed three times to provide a total of 18 RNA samples. RNA quantitation and quality control was performed on a Fragment Analyzer (Advanced Analytical) and cDNA libraries were constructed by standard methods (Illumina TruSeq mRNA stranded kit) with index adapters (Illumina TruSeq indexes). DNA was then sequenced as single-end, 50 base-length reads on a NovaSeq6000 instrument (Illumina, Inc.) with an average read count of 24-34 million reads per sample. Initial base calling and quality filtering of the mRNA-seq reads generated with the Illumina analysis pipeline (fastQ format) were performed with the FastxToolkit (http://hannonlab.cshl.edu/fastx_toolkit/) and adapters trimmed by using CutAdapt (https://cutadapt.readthedocs.org/en/stable/). Reads that mapped either to the human mitochondrial genome or to the internal Phix standard were removed through use of Bowtie software (http://bowtie-bio.sourceforge.net/index.shtml). Reads were mapped (TopHat v2.0.13; http://ccb.jhu.edu/software/tophat/index.shtml) to the reference genome (from Ensembl, v78; http://www.ensembl.org/index.html; Homo_sapiens.GRCh38.dna.primary assembly.fa with Homo_sapiens.GRCh38.78.gtf as annotation). Tests for differential expression were made by using cuffdiff v2.2.1. Uniquely mapped reads were counted with htseq-count v.011.1. Gene expression levels were calculated in FPKM (fragments per kilobase of exon per million mapped fragments), considering gene length as a sum of all exonic nonoverlapping sequences of all isoforms of a given gene. Regularized log transformed (rlog) counts output from DESeq2 to plot heatmaps and gene expression. Tests for differential expression (q-value < 0.01, Log2(foldchange) > 1.5; the Benjamini–Hochberg P value adjustment method was used with DESeq2 within Rstudio (RStudio, Inc.). To broadly analyze pathways affected by *VTCN1* suppression, regulated genes with identifiable alterations upon *VTCN1* knock-down were further analyzed by KEGG pathway enrichment with the web-based DAVID tool (Database for Annotation, Visualization, and Integrated Discovery v6.8). Further clustering and pathway analyses were performed by Ingenuity Pathway Analysis (Qiagen, Hilden, Germany).

### Immunostaining

Cells were grown on coverslips coated with Matrigel and placed in six-well tissue culture plates as described previously (19). After fixing the cells in 4% paraformaldehyde (PFA) in PBS for 10 min and permeabilizing them in 1.0% Triton X-100/PBS for 30 min, coverslips were placed in 5% (vol/vol) goat serum/5% (wt/vol) BSA in PBS for 1 h. Cells were then incubated with appropriately diluted primary antibodies (**Table 2**) overnight at 4°C. Secondary antibody staining was performed with either Alexa Fluor 568-, 647-, or 488-labeled detection antibodies at a 1:300 dilution for 2 hours at room temperature (**Table 2**). Images were captured under a Zeiss Axiovert 200M with a Leica DFC290 color camera.

**Table 2:** Primary and secondary antibodies used in immunostaining.

Antibody	Catalog no. (Source)	Dilution
B7H4	Ab209242(Abcam)	1:100
CGA	MAB4169(R&D)	1:100
CGB	Ab53087 (Abcam)	1:100
HLA-A	Ab52922(Abcam)	1:100
HLA-A	A2167 (ABclonal)	1:100
HLA-B (A, C)	Ab225636(Abcam)	1:100
HLA-B	A1285 (ABclonal)	1:100
HLA-C	Ab126722(Abcam)	1:100
HLA-G	sc-2179 (Santa Cruz Biotechnology)	1:50
Alexa Fluor 488 donkey anti-mouse IgG	A-21202 (Life Technologies)	1:300
Alexa Fluor 488 donkey anti-rabbit IgG	A-21208 (Life Technologies)	1:300
Alexa Fluor 555 donkey anti-mouse IgG	A-31570 (Life Technologies)	1:300

### Western blotting

Proteins were extracted from cells in radioimmunoprecipitation assay (RIPA) buffer [10 mM Tris·HCl (pH 7.2), 1 mM EDTA, 1% Triton X-100, 0.1% SDS, 0.1% sodium deoxycholate, and 100 mM NaCl], then fractionated by SDS-PAGE and transferred to a polyvinylidene difluoride membrane by means of a transfer apparatus according to the manufacturer’s protocols (Bio-Rad). After incubation with 5% nonfat milk in Tris-buffered saline/Tween 20 (TBST; 10 mM Tris, pH 8.0, 150 mM NaCl, 0.5% Tween 20) for 60 min, the membrane was washed once with TBST, and target proteins detected by incubating with the primary antibodies described in **Table 3** at 4°C for 12 h. Membranes were washed three times for 10 min and incubated with a 1:3000 dilution of horseradish peroxidase-conjugated anti-mouse or anti-rabbit antibodies for 2 h. Blots were washed with TBST three times and developed with the UVP imaging system (AnalytikJena) according to the manufacturer’s protocols. Pairwise comparisons were made with the Student’s t test. Values of p<0.05 were considered to support the conclusion that differences were statistically significant.

**Table 3:** Primary and secondary antibodies used in western blotting.

Antibody	Catalog no. (Source)	Dilution
B7-H4	14572(Cell Signaling Technology)	1:1000
HLA-A	Ab52922(Abcam)	1:1000
HLA-A	A2167 (ABclonal)	1:1000
HLA-B (A, C)	Ab225636(Abcam)	1:1000
HLA-B	A1285 (ABclonal)	1:1000
HLA-C	Ab126722(Abcam)	1:1000
HLA-G	sc-2179 (Santa Cruz Biotechnology)	1:500
MAPK	4695 (Cell Signaling Technology)	1:1000
pMAPK	4370 (Cell Signaling Technology)	1:1000
pSTAT1	9177 (Cell Signaling Technology)	1:1000
IFITM1	13126 (Cell Signaling Technology)	1:1000
GAPDH	5174 (Cell Signaling Technology)	1:2000

### Invasion assay

An aliquot of 5 x 10^4^ H1 cells was transferred to invasion chamber wells as previously described (21). After 24 h from initial plating, the medium was changed (2 ml in each chamber) to BAP treatment for differentiation. The medium was then changed daily for 3 days. On the third day (4 days after initial plating), the cells were transfected with *VTCN1* knock-down or scrambled-control siRNA for 6 h. Then the medium was replaced with BAP treatment medium and the cells cultured for another 3 days. On the sixth day the cells on the lower surface of the membrane were fixed in 4% PFA and then stained with DAPI (diluted in PBS, 1:750). Each membrane was imaged by taking 12 images per membrane at 10 x magnification and the number of nuclei counted by means of Image J software. Pairwise comparisons were determined using the Student’s t test, with a significance of p<0.05 indicated by asterisks.

### Immunoassays

After 6 days of BAP treatment with siRNA transfection as above, spent culture medium was collected to measure hCG (human chorionic gonadotrophin) levels (Genway Biotech, LLC; GWB-BQK0F2). Separate but identically treated wells had spent culture medium collected on culture days 7 and 8. Total DNA was isolated (Promega, #A2360) from the respective cultures in order to normalize immunoassay results to cell density levels. ELISAs were performed by following the manufacturer-recommended protocols. Samples were collected in three independent experiments for each treatment. Data followed over time were subjected to two-way ANOVA, followed by the Bonferroni test for pairwise comparisons. Values of p<0.05 were considered to support the conclusion that differences were statistically significant.

## Results

### The inhibitory T cell co-receptor molecule, *VTCN1*, promotes syncytialization and inhibits invasion in BAP cells

**Figure 2:**
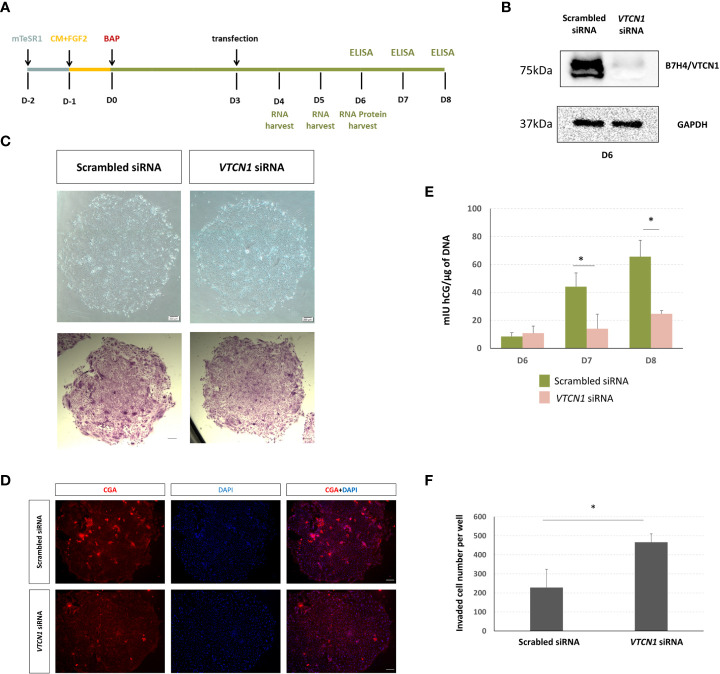
**(A)** Human ESCs (H1) were maintained on mTeSR1. Following the day of passaging, medium was changed to MEF-conditioned medium supplemented with FGF2 (4 ng/µL) (CM+FGF2). After an additional day, the medium was replaced with DME/F12 and 20% KOSR supplemented with BMP (10 ng/mL), A83-01 (1µM), and PD173074 (0.1 µM) (DME/F12/KOSR/KOSR+BMP4/A83/PD) for 8 d. BAP treated H1 ESC were transiently transfected with VTCN1 siRNA or scrambled siRNA (10nM) for 3 d beginning on d3 of BAP treatment. **(B)** VTCN/B7H4 protein expression was assessed by western blotting on BAP treatment d6 (3 days after transfection). **(C)** Phase contrast images of cell colonies on d6 (top). Cells were fixed and stained with crystal violet on D6 and images captured under bright field (bottom). **(D)** In separate experiments, VTCN1 KD and scrambled control cells were fixed on d6 of BAP treatment and stained for CGA (Scale bar, 100 µm). **(E)** Daily (24 h) hCG production was assessed by using ELISA on d6, d7 and d8 following initiation of BAP-induced differentiation and VTCN1 KD on d3 of differentiation. Protein concentrations by ELISA were normalized to the DNA content of each culture. Values are means ± SD for the individual experiments. The VTCN1 siRNA treated cultures secreted significantly lower levels of hCG by d7 and d8 when compared to controls (p=0.025 and p=0.04, respectively). **(F)** Comparison of TB invasion in BAP treated H1 ESC transfected with VTCN1 siRNA or scramble siRNA for 3 days. Values are the mean number of invaded cells ± SD in 12 randomly selected fields for three independent experiments, each in triplicate wells. Statistically significant comparisons to scrambled control siRNA are indicated by asterisks (p<0.05).

H1 (WA01) ESCs were differentiated to TB with BMP4, A83-01, and PD173074 (BAP treatment) as previously described for up to 8 days (10 total days in culture) (19). Total protein was collected from BAP cells on treatment days 3 through 8 (**Figure 2A**). Western immunoblotting demonstrated marked increases in *VTCN1* in BAP treated cells beginning 4 days after exposure and continuing through d8 (10 days of culture) (**Supplementary Figure 1A**).

To determine possible roles for *VTCN1* in general TB development and function, we used siRNA to block *VTCN1* transcription (**Figure 2A**). *VTCN1* mRNA knock-down (KD) was confirmed by qPCR and decreases in protein expression demonstrated by western blotting and immunofluorescence histochemistry (IHC, **Figure 2B**, **Supplementary Figures 1B-D**). We began our analyses on the effects of *VTCN1* KD by assessing typical BAP-induced TB developmental milestones. To determine whether *VTCN1* regulated the syncytialization of TB cells, we transfected BAP cells with *VTCN1*-specific and scrambled control siRNA on treatment d3 (**Figure 2A**). Direct phase contrast imaging and crystal violet staining, which preferentially highlights syncytialized patches of cells within the colonies, demonstrated impairment of syncytialization in *VTCN1* siRNA- but not in scrambled control siRNA-exposed cells (**Figure 2C**). Fluorescence immunohistochemistry was used to assess the effects of *VTCN1* suppression on two protein markers of TB cell syncytialization, CGB and CGA (**Supplementary Figure 1E**, **2D**, respectively). *VTCN1* KD did not affect CGB expression on BAP d6, which was expected, as CGB expression in BAP cells is typically first detected later in the differentiation progression (19). In contrast, CGA expression, which typically increases in the days prior to the initiation of syncytialization in BAP cultures, decreased upon *VTCN1* KD (**Figure 2D**), consistent with the visual reductions in STB areas within colonies discussed above (**Figure 2C**).

We next used time-course experiments beginning on d6 of BAP treatment (3 days after *VTCN1* KD) to document the effects of *VTCN1* KD on secretion of hCG, whose concentrations were normalized to DNA content of the respective cultures to adjust for possible treatment-related differences in cell growth and proliferation. Confirming the IHC results, BAP treated TB cells exposed to *VTCN1* siRNA produced similar amounts of hCG on d6 when compared to scrambled siRNA controls (**Figure 2E**).

The noted decrease in syncytialization imparted by *VTCN1* KD led us to hypothesize that the BAP cells were differentiating through an alternative developmental pathway, most likely conversion into invasive EVT-like cells (22). In experiments replicating the BAP and transfection methods described above, BAP treated TB cells were exposed to *VTCN1* or scrambled control siRNA but were then cultured under conditions that allowed specific assessment of TB cell invasion (invasion assays). The invasive capacity of BAP cells increased after *VTCN1* KD when compared to scrambled siRNA-transfected cells (p=0.032) (**Figure 2F**).

### The interferon response pathway is activated upon *VTCN1* repression in TB cells

We next performed RNA-seq on BAP cells at 24, 48 and 72 h after *VTCN1* KD to define pathways regulated by *VTCN1* better. A total of 61 genes were up-regulated and 155 were down-regulated 24 h after transfection. At this time point, 21 up-regulated pathways were enriched with a criterion of p<0.05 while no down-regulated pathways were enriched (**Supplementary Figure 2A**, **Supplementary Table 1**). Upregulated, differentially expressed genes at the 24 h time point were highly associated with pathways that included: herpes simplex infection, influenza A and cytokine receptor interaction. Of the 21, 20 were associated with immune responses. Analyses of the 48 h and 72 h post-transfection samples identified a further 12 and seven upregulated and enriched pathways, respectively (**Supplementary Figure 2B, C**, **Supplementary Table 1**), with upregulated differentially expressed genes highly associated with pathways that again included responses to virus.

**Figure 3:**
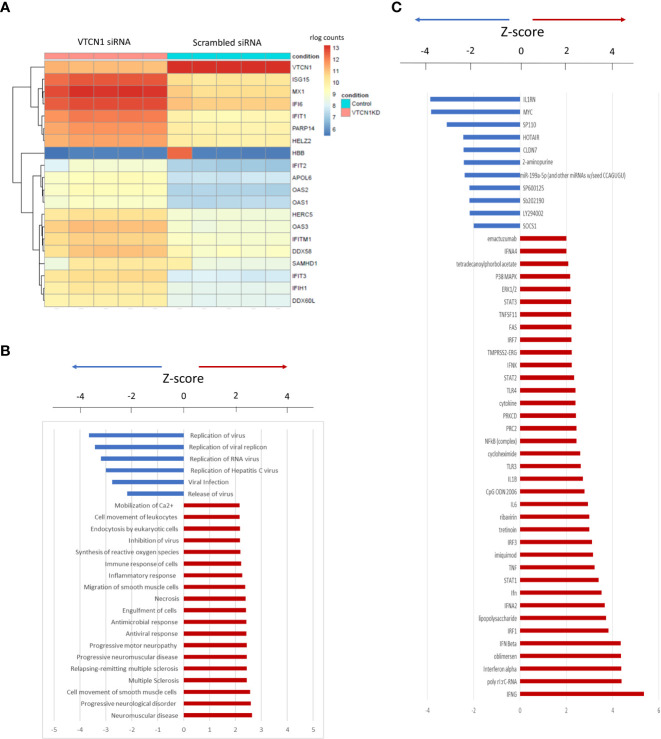
**(A)** Heatmap of the top 20 differentially expressed genes 24 h after VTCN1 KD on d3 of BAP treatment. rlog count (regularized logarithm transformed count) reflects gene expression normalized to the sequencing depth of each sample. **(B)** Affected functional categories 24 h after transfection. **(C)** Gene activation 24 h after transfection.

To assess the effect of *VTCN1* KD at the single gene level in more detail, we generated a clustering heatmap of differentially expressed genes. The top 20 genes identified in this analysis are shown in the heatmap in **Figure 3A**, among which 16 genes are related to type I IFN responses, which are known to mediate innate immune responses to viruses. The genes most altered by *VTCN1* suppression when compared to scrambled siRNA exposed controls were next analyzed by using QIAGEN Ingenuity Pathway Analysis software (QIAGEN IPA). The affected functional categories with highest significance 24 h, 48 h and 72 h after transfection are presented in **Figure 3A, B**, **Supplementary Figure 3A** and **Supplementary Figure 4A**. Potential upstream regulators of the differentially expressed genes as predicted from the literature by IPA (Ingenuity Pathway Analysis) are shown in **Figure 3C**, **Supplementary Figure 3B** and **Supplementary Figure 4B**.

### Signaling pathways involved during the promotion of invasion

**Figure 4:**
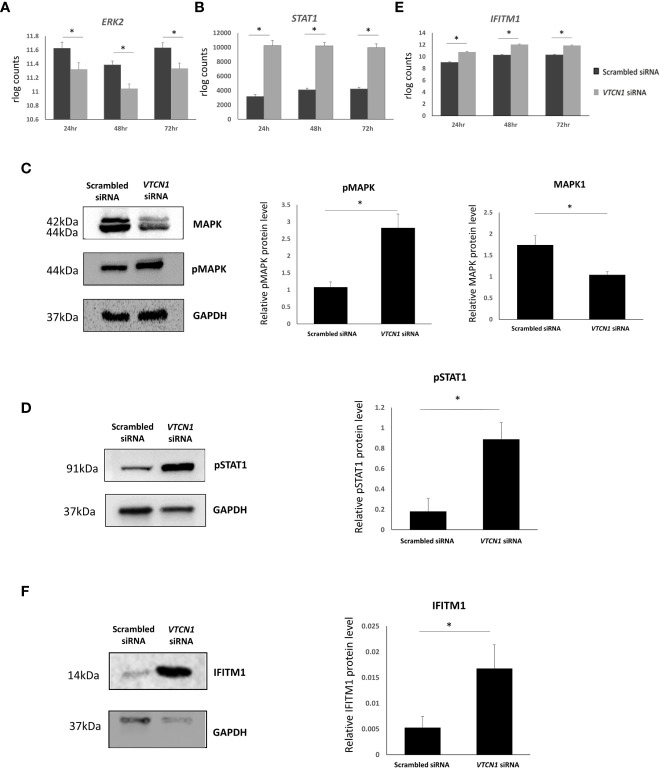
Transcript expression levels (in rlog counts) for ERK2 **(A)**, STAT1 **(B)**, and IFITM1 **(E)** in BAP differentiated TB cells exposed to scrambled siRNA or VTCN1 siRNA for 24h, 48h and 72h (BAP d4, d5 and d6). The hESCs were passaged and transfected as explained in the legend of **Figure 1**. Benjamini–Hochberg P value adjustment method were done with DESeq2. An adjusted p value less than 0.01 was considered to be statistically significant. Protein levels of MAPK and pMAPK **(C)**; pSTAT1 **(D)**; and IFITM **(F)** in BAP differentiated TB cells on d6 (3 days after transfection) were assessed by western blotting. Three independent western blots were quantified by densitometry using ImageJ Software. The protein expression of MAPK, pMAPK, pSTAT1 and IFITM1 was normalized to the corresponding GAPDH signals of appropriate samples. Pairwise comparisons were determined using the Student’s t test, with a significance of p < 0.05 indicated by asterisks.

RNA-seq data rlog counts normalized to scrambled siRNA controls demonstrated a decrease in *MAPK* and an increase in *STAT1* transcripts in response to *VTCN1* siRNA knock-down (**Figures 4A, B**). Additionally, the expression of EVT pro-invasion markers, Integrin Subunit Alpha 5 (ITGA5) and matrix metalloproteinases (MMP)-12, were significantly increased after *VTCN1* had been knocked down (**Supplementary Table 2**). To verify the documented transcriptional changes seen with bulk RNA-seq as well as activation of the MAPK/ERK1/2 and JAK/STAT signaling pathways upon *VTCN1* KD, protein levels of MAPK, phospho-MAPK (pMAPK) and phospho-Stat1 (pSTAT1) were examined by western blotting. As with the transcriptional changes in *STAT1*, pSTAT1 level increased in TB cells upon *VTCN1* KD (**Figure 4D**). While the direction of change in pMAPK protein expression upon VTCN1 KD followed that of the transcriptional changes, protein levels of pMAPK, using an antibody detecting p44/42 MAPK, were significantly elevated (**Figure 4C**).

### IFITM1 overexpression inhibits TB cell fusion

RNAseq data from *VTCN1* KD cells also highlighted an increase in *IFITM1* transcripts when compared to scrambled control siRNA exposed cells. (**Figure 4E**). The IFN-induced transmembrane protein (IFITM) family includes members (IFITM1, -2, and -3) that protect multiple cell types from viral infections by preventing viral membrane fusion with cells and by inhibiting the syncytialization of infected cells (23–25). Since syncytialization is central to TB development, we compared IFITM1 protein dynamics by Western blotting to placental TB developmental changes in BAP primed TB with or without *VTCN1*. As expected, IFITM1 increased as syncytialization decreased when *VTCN1* was knocked down in BAP cells (**Figure 4F**).

### Transcription and translation of classical MHC class I molecules increase with *VTCN1* repression in BAP cells

**Figure 5:**
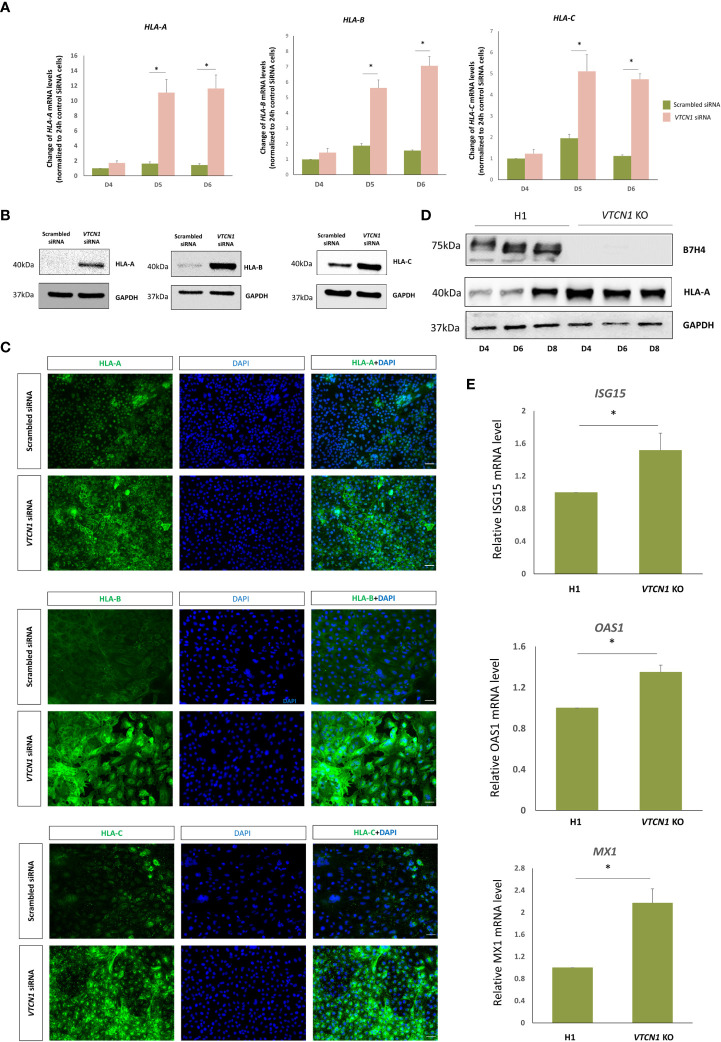
BAP treated H1 ESC were transiently transfected with VTCN1 siRNA or a scrambled siRNA control for 3d. **(A)** The effects of VTCN1 KD on HLA-A, HLA-B and HLA-C transcription were assessed by RT-PCR. Data represents means ± SD of three independent experiments; statistically significant comparisons to scrambled control siRNA are indicated by asterisks (p<0.05). **(B)** Protein was collected on BAP d6 (transfection d3) and the expression of HLA-A, HLA-B and HLA-C were assessed by western blotting **(C)** Immunofluorescence microscopy was performed to assess the expression of HLA-A, HLA-B and HLA-C on BAP d6 (transfection d3) (Scale bar, 100 µm). **(D)** Western blotting analysis of HLA-A protein expression in BAP treated H1 cells or VTCN1 KO cells on BAP d4, d6 and d8. **(E)** ISG15, OAS1 and MX1 mRNA levels for BAP treated H1 cells (WT) and VTCN1 KO cells were assessed by qPCR on BAP d6.

**Figure 6:**
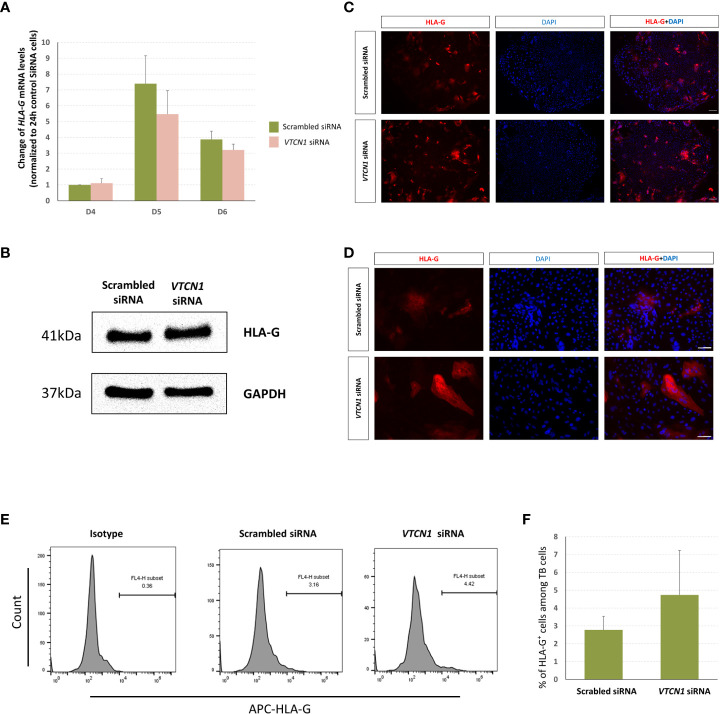
H1 ESC on D3 of BAP differentiation were transiently transfected with VTCN1 siRNA or a scrambled siRNA control for 3 d, **(A)** HLA-G mRNA was assessed by RT-PCR. Data represents means ± SD of three independent experiments; pairwise comparisons were made using Bonferroni methods. No statistically significant differences were detected in HLA-G transcripts in VTCN1 KD cells when compared to scramble siRNA transfected cells. **(B)** Protein was collected on BAP d6 (KD d3) and the expression of HLA-G was assessed by western blotting. **(C, D)** Immunofluorescence microscopy was performed to assess the expression of HLA-G on BAP treatment d6 (KD d3) (Scale bar, 100 µm). **(E, F)** Cells were collected on BAP treatment d8 (KD d5) and flow cytometry was used to track the cell surface expression of HLA-G.

RNA-seq analyses provided evidence that the mRNA levels of classical MHC class I molecules, *HLA-A*, *HLA-B* and *HLA-C* increase with *VTCN1* suppression, while *HLA-G* transcripts remain relatively unchanged. The changes in *HLA-A*, *-B*, *-C* and *-G* expression were validated by RT-PCR and correlated well with protein data acquired by western immunoblotting, fluorescence ICC and flow cytometry (**Figures 5**, **6**). *HLA-A*, *HLA-B* and *HLA-C* transcripts increased on d5 and d6 of BAP treatment (d2 and d3 post *VTCN1* KD) (**Figure 5A**) and protein expression increased on BAP d6/*VTCN1* KD d3 (**Figures 5B, C**). The effects of *VTCN1* KD on the transcript and protein levels of *HLA-G*, a marker for EVT, were similarly analyzed and were not affected by treatment.

To identify possible off target gene silencing during siRNA treatment and/or the possibility of a purely interferon-stimulated increase in classical MHC class I expression secondary to a cellular response to the siRNA, we monitored HLA-A protein levels and IFN-stimulated genes expression in a *VTCN1* CRISPR-Cas9 knock-out (KO) hESC cell line after BAP treatment. HLA-A protein on d4, d6 and d8 of BAP treated *VTCN1* KO cells and IFN-stimulated genes including *ISG15*, *OAS1* and *MX1* mRNA levels on d6 of BAP treated *VTCN1* KO cells increased when compared to BAP-exposed wild type H1 cells (**Figures 5D, E**), replicating findings in our siRNA-induced transient knock-down models (**Figure 3A**, **Figures 5A-C**).

To further verify a relationship between *VTCN1* and *MHC class I* gene alterations during primitive TB differentiation, we used an alternative model and analyzed a publicly-available single cell RNA-seq database generated by West et al. for TB cells isolated from embryonic day (D) 8, D 10 and D 12 human embryos during blastocyst extended culture (26). Although the transcripts for *VTCN1*, *HLA-A* and *HLA-B* were all expressed at low levels in cytotrophoblast (CTB) at D 8, D 10 and D 12, the expression of *VTCN1* progressively decreased as the expression of *HLA-A* and *HLA-B* increased over time in culture, suggesting a possible association between B7-H4 and MHC class I during early placental development. In contrast, *HLA-C* and the non-classical MHC I molecules *HLA-E* and *HLA-G* genes were more robustly expressed throughout culture, and expression increased dramatically from D 10 to D 12 in culture (**Supplementary Figure 5**), likely a reflection of an increasing proportion of cells that are migratory (EVT-like) in the embryos.

### *VTCN1* in BAP cells shifts the phenotype of co-cultured peripheral NK cells towards that of decidual NK cells

**Figure 7:**
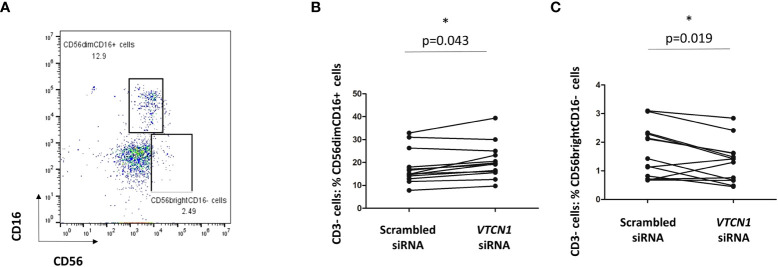
**(A)** PBMCs isolated from pregnant volunteers were co-cultured with TB cells with or without VTCN1 KD from BAP d5 to d8 (KD d2-d5). Flow cytometry was used to analyze the CD56 and CD16 expression on CD3- lymphocytes in co-culture. **(B, C)** Flow cytometry was used to analyze the effects of VTCN1 KD in TB cells on human CD3- CD16+ CD56dim cells **(B)** and CD3- CD16- CD56bright **(C)** peripheral blood cells after coculture with TB cells. Results are expressed as percentages. p-values were calculated using the paired t-test (n=13); significant differences are indicated by asterisks.

We hypothesized that alterations in MHC cell surface expression in invasive primitive trophoblast of the implanting embryo might play a role in possible phenotype switching of peripheral maternal CD3-CD56^dim^CD16^+^ NK cells toward the CD3^-^CD56^bright^CD16^-^ NK cells typical of the decidua (27). Since *VTCN1* KD increased the surface expression of classical MHC class I molecules, including HLA-C, on BAP primed TB cells, we examined the effects of such changes on peripheral immune cells from pregnant women in a co-culture system. Cryopreserved PBMCs isolated from peripheral blood of pregnant women (5-12 weeks gestation) were thawed and cocultured for 3 days (d5 – d8 of BAP differentiation/d3–d5 post *VTCN1* KD) with control and *VTCN1* KD BAP-treated human ES cells. Non-adherent cells in the co-culture, largely PBMCs, were then isolated and subjected to flow cytometry. Lymphocytes were identified by FSC/SSC light scatter. The gating strategy for CD56 and CD16 expression on all CD3-negative cells is presented in **Figure 7A**. The percentage of CD3-CD56^dim^CD16^+^ peripheral-type NK cells among CD3 negative lymphocytes increased (p=0.043, **Figure 7B**) and the percentage of CD3^-^CD56^bright^CD16^-^ decidual NK-like cells decreased (p = 0.019, **Figure 7C**) in PBMCs cocultured with *VTCN1* KD TBs when compared to those cocultured with scrambled control siRNA-exposed TB.

## Discussion

We previously demonstrated that *in vivo* protein expression of the B7 family checkpoint inhibitor, *VTCN1* (B7-H4), is at its highest in villous trophoblast during the first few weeks of pregnancy (11). It is also expressed in trophoblast differentiated from pluripotent stem cells that we believe may correspond to primitive trophoblast associated with the implanting conceptus (10, 11). *VTCN1* is known to mediate immune homeostasis at several sites of inflammation, including those in the tumor microenvironment (28), and we hypothesized that it might also play an analogous role in maternal-fetal immune regulation. Here, we’ve studied the role of *VTCN1* and its translation product in early human placental development and in immunologic interactions at the human maternal-fetal interface.

Downregulation of *VTCN1* expression in our *in vitro* model of peri-implantation human TB development shifted cells away from syncytialization (**Figures 2B, E**) and toward invasion (), consistent with findings in some cancers. The cancer literature contains inconsistent findings on the associations between *VTCN1* (12, 13) and metastatic behaviors. High levels of *VTCN1* have been observed in invasive breast cancer (13), and high tumor cell expression has been linked to disease progression and poor prognosis in renal cell cancer (12), suggesting that *VTCN1* promotes tumor invasion. On the other hand, high levels have been positively correlated with improved survival in breast cancer patients and smaller tumors in a B7-H4 ^-/-^ murine model of breast cancer (29), seemingly supporting a converse relationship with invasion. Our study supports the former role in the human placenta. We suggest that *VTCN1* may limit invasion and promote syncytialization at the earliest stages of gestation but permit a shift toward invasion as pregnancy progresses and *VTCN1* expression decreases. This early bias toward STB lineage development is consistent with the essential role of hCG, a primary secretory product of STB, in the support of early pregnancy when the conceptus must signal the mother to avoid a return to ovarian cyclicity. It is also consistent with the observation that peak levels of hCG occur during the first trimester and subsequently decline (30, 31). The shift away from syncytialization and toward invasion upon *VTCN1* suppression in our model system was not associated with a simultaneous change in HLA-G expression, a marker of the invasive trophoblast subtypes. It is possible that changes in *VTCN1* expression uncouple invasion/migration and HLA-G expression.

To investigate *VTCN1*-mediated inhibition of invasion in the human placenta, we assessed the effect of *VTCN1* knock-down on signaling pathways known to be central to the maintenance and/or differentiation of human TB subtypes (32). Activation of the MAPK/ERK1/2 pathway promotes invasion in the transformed human cell line HTR-8/SVneo, which models the EVT lineage (33), while inhibition of STAT1 limits invasion in HTR‐8/SVneo and JEG‐3 cells (34, 35). The latter are commonly used to model CTB proliferation and invasion (36). We demonstrate that *VTCN1* knock-down activates the MAPK/ERK1/2 and JAK/STAT pathways and increases BAP-primed TB invasion (**Figure 2F**, **Figures 4C, D**). Although these same signaling pathways are also reported to regulate TB cell fusion in primary term TB cell cultures and during forskolin-mediated syncytialization in BeWo cells (37, 38), upregulation of pMAPK and pSTAT1 by *VTCN1* knockdown was not associated with spontaneous cell fusion in our study.

IFN-inducible transmembrane 1 (IFITM1) expression was significantly increased upon *VTCN1* KD in our model of primitive TB development ((**Figure 3A**, **Figures 4E, F**; also see below). Although IFITM1 is known to protect uninfected cells from viral infection *via* blocking virus-cell fusion and opposing entry by many enveloped viruses (24, 25, 39), IFITM1 also impairs syncytin-mediated fusion (23, 40). We have shown previously that IFN responses are blunted in primitive TB when compared to term TB counterparts (41), so the increase in IFITM1 upon knockdown of *VTCN1* in our model is consistent with this. We hypothesize that the increase in IFITM1 upon *VTCN1* knockdown in primitive TB may account, at least in part, for the reduction in STB formation.

During pregnancy, there is a shift from the marked inflammatory attachment reaction at the initiation of pregnancy (implantation) to a more pro-tolerogenic, non-inflammatory state for the majority of the remainder of pregnancy (1, 42, 43). Unlike TB derived from term placenta, BAP-generated primitive TB of the early first trimester has diminished levels of 1) interferon-stimulated genes (ISG), such as *ISG15*, *MX1*, *DDX58* (*RIG-*1), 2) interferon induced proteins with tetratricopeptide repeats (*IFIT*) family members, 3) and oligoadenylate synthase-like (*OAS*) family members, all of which augment the innate immune response to viral infection (44, 45). Such diminished responses have been linked to a greater risk of vertical transmission of viruses in early gestation including transmission of CMV, HSV, Zika virus and SARS-CoV-2 (46–50). Our RNA-seq analysis revealed that expression of several ISGs were upregulated when *VTCN1* expression was repressed (**Figure 3A**). Pathway enrichment and clustering analyses indicated that *VTCN1* expression negatively regulates innate immune response and inflammation in hESC primed TB. These activities uphold its known role as an immune checkpoint regulator but might also confer on early pregnancy an increased susceptibility to virus infection (**Figure 3A**, **Supplementary Figure 2**-**4**), which is consistent with our prior findings (41, 44).

We also demonstrated that transcription and translation of classical major histocompatibility complex (MHC) class I molecules are increased in TB when *VTCN1* expression is knocked down (**Figure 5**). MHC expression patterns in the human placenta are unique, and the control of MHC class I expression is poorly understood (51–54). Villous CTB and STB do not express the classical MHC class I molecules, HLA-A, -B and -C. EVT also lack HLA-A and -B, but express HLA-C and several non-classical MHC class I molecules, including HLA-G (48, 53–55). We have shown that *VTCN1* knock-down upregulates mRNA and protein levels of HLA-A, -B and -C, but has little effect on HLA-G expression. These findings are supported by TB single-cell RNA-seq data derived from human blastocysts grown *in vitro* for up to 12 days post-fertilization (extended blastocyst culture) (26). These embryos allow the first 5 days of placental growth to occur in the absence of maternal signals. In such an extended human blastocyst culture model, low *VTCN1* expression correlated with an elevation in classical MHC-I expression in early TB cells (**Supplementary Figure 5**). Together, these data are consistent with an extensive literature demonstrating that *VTCN1* is expressed in a large variety of malignancies (12–14, 55) and that MHC class I products are simultaneously often poorly expressed in many (estimates range from 40-90%) of such tumors (56–58), thereby enhancing evasion of immune responses by the host.

Since HLA-A, -B and -C (but not HLA-G) expression is responsive to IFNA and -G exposure (59, 60), and siRNAs are notorious for causing off-target antiviral IFN responses, we determined whether similar changes were observed when CRISPR-Cas9 was used to achieve a biallelic knockout of all variants of *VTCN1* in undifferentiated H1 cells. Again, increases in MHC class I expression and modest but significant upregulation of certain interferon-inducible genes were noted during BAP-induced differentiation of the mutant H1 cells, suggesting that the changes observed after siRNA treatment were not artifactual.

While it is clear that human decidual NK cells have marked phenotypic and functional differences from their peripheral counterparts (61–72), what remains less clear is their origin, with some scholars suggesting they derive from expansion of an *in situ* endometrial population (61, 62, 73, 74) and others suggesting many are called to the site of implantation from a pNK cell population and either expanded or differentiated in the local microenvironment (63–67). It is likely that both are involved. In this study, we have reported that the presence and absence of TB-expressed *VTCN1* can shift the phenotype of peripheral NK cells isolated from pregnant women from the CD56^dim^CD16^+^, classically cytotoxic subtype (68, 69) to the CD56^bright^CD16^dim^ subtype more typical of the decidua (63–68). This finding suggests that the expression of the immune checkpoint regulator, B7-H4, on the surface of TB could be involved in local differentiation of pNK cells, possibly those called into the decidua in response to the hormonal changes of ovulation and corpus luteum formation, toward a decidual phenotype. While changes in surface MHC expression may be involved,. parthenogenesis embryonic stem cells and animal experiment will be conducted to dissect the machanisms underlying this phenotypic shift and its role in the nuanced innate and adaptive immune interaction at the maternal-fetal interface of early human pregnancy ( (70, 71)).

The BAP-derived TB and extended human blastocyst culture systems have recognized limitations. Still, as two of a very limited number of approaches to the study of the earliest stages of human pregnancy, their concordant results around *VTCN1* are reassuring. Recently, the derivation, culture and differentiation conditions for human trophoblast stem cells (hTSCs) were reported (72) and hTSC were subsequently generated from pluripotent stem cells including hESC and iPSC (75–82). Future experiments will utilize hTSC-based models to differentiate among trophoblast sublineages, co-culture systems that allow study of TB interactions with maternal decidual and vascular cells and expanded study of the *VTCN1* CRISPR-Cas9 knock-out (KO) hESC cell line. Future investigation on *VTCN1*-related changes in NK cells will also be expanded to analyze NK cell function in addition to the phenotypic changes reported her.

In summary, we suggest that: 1) VTCN1 is a critical regulator of TB syncytialization, TB invasiveness and possibly other aspects of differentiation in the early human placenta; 2) *VTCN1* limits upregulation of classical MHC Class I genes and an array of proteins involved in interferon responses in TB, including that of IFITM1, a protein whose expressions counteracts syncytialization; and 3) *VTCN1* presentation by TB cells can induce phenotypic changes in peripheral natural killer cells that resemble those characteristics of the maternal-fetal interface. In sum, the immune checkpoint regulator, *VTCN1*, likely plays an important role in early placental development and therefore diseases of abnormal placentation.

## Data availability statement

The original contributions presented in the study are included in the article/**Supplementary Material**. Further inquiries can be directed to the corresponding author.

## Ethics statement

The studies involving human participants were reviewed and approved by University of Missouri, Columbia, Office of Human Research Protection Program, Medical IRB Committee-1 #2017804. The patients/participants provided their written informed consent to participate in this study.

## Author contributions

JZ and DS designed research. JZ, YT and MW performed research. YQ conducted the bioinformatics analyses. JZ performed statistical analyses. JZ and DS analyzed data. DS recruited patients. JZ and DS wrote the manuscript. All authors contributed to the article and approved the submitted version.

## Supplementary material

Supplementary Figure 1
**(A)** Time course for protein expression of B7H4 in BAP treated H1 hESCs from BAP d3 to d8. **(B)** BAP treated H1 hESCs were transiently transfected with scramble siRNA (control) or VTCN1 siRNA (VTCN1 KD) and cultured for up to 72 h. Assessments were made 24 h, 48 h and 72 h after transfection. Relative concentrations of VTCN1 transcripts were assessed by real-time PCR (n=3; i.e., three RNA preparations from three independent experiments). Data represents means ± SD, statistically significant values compared to scrambled control siRNA-exposed cells are indicated by asterisks (p<0.05). **(C–E)** BAP treated H1 hESCs were transiently transfected with scramble siRNA (control) or VTCN1 siRNA (VTCN1 KD) and kept in culture for 3 d after transfection. B7H4 **(C, D)** and CGB **(E)** protein expression was assessed by immunofluorescence microscopy (Scale bar, 100 µm).

Supplementary Figure 2 KEGG pathway analysis of differentially expressed genes after exposure to VTCN1 siRNA and a matched scrambled siRNA control. **(A)** Genes upregulated 24 h after transfection, **(B)** genes upregulated 48 h after transfection, **(C)** genes upregulated 72 h after transfection.

Supplementary Figure 3 Assessment of gene activation patterns for differentially expressed genes after exposure to VTCN1 siRNA and a matched scrambled siRNA control. **(A)** Affected functional categories 48 h after transfection. **(B)** Activated genes 48 h after transfection.

Supplementary Figure 4 Assessment of gene activation patterns for differentially expressed genes after exposure to VTCN1 siRNA and a matched scrambled siRNA control. **(A)** Affected functional categories 72 h after transfection. **(B)** Activated genes 72 h after transfection.

Supplementary Figure 5 Data were generated in a model of human extended blastocyst culture (26, 83) and were analyzed by using the publicly-available databases generated from that work. **(A-F)** MHC-I expression in early pregnancy. FPKM values of the MHC-I genes: HLA-A **(A)**, HLA-B **(B)**, HLA-C **(C)**, HLA-E **(D)**, HLA-G **(E)** and of VTCN1 **(F)** in small cells of human embryos on d8, d10 and d12. The authors suggest these small cells represent mononucleated CTB.
