# Supplementary figures and images for: The immune checkpoint molecule, VTCN1/B7-H4, guides differentiation and suppresses proinflammatory responses and MHC class I expression in an embryonic stem cell-derived model of human trophoblast
Source: Front Endocrinol (Lausanne). 2023 Mar 16;14:1069395. doi: 10.3389/fendo.2023.1069395 (PMC10062451; doi:10.3389/fendo.2023.1069395)

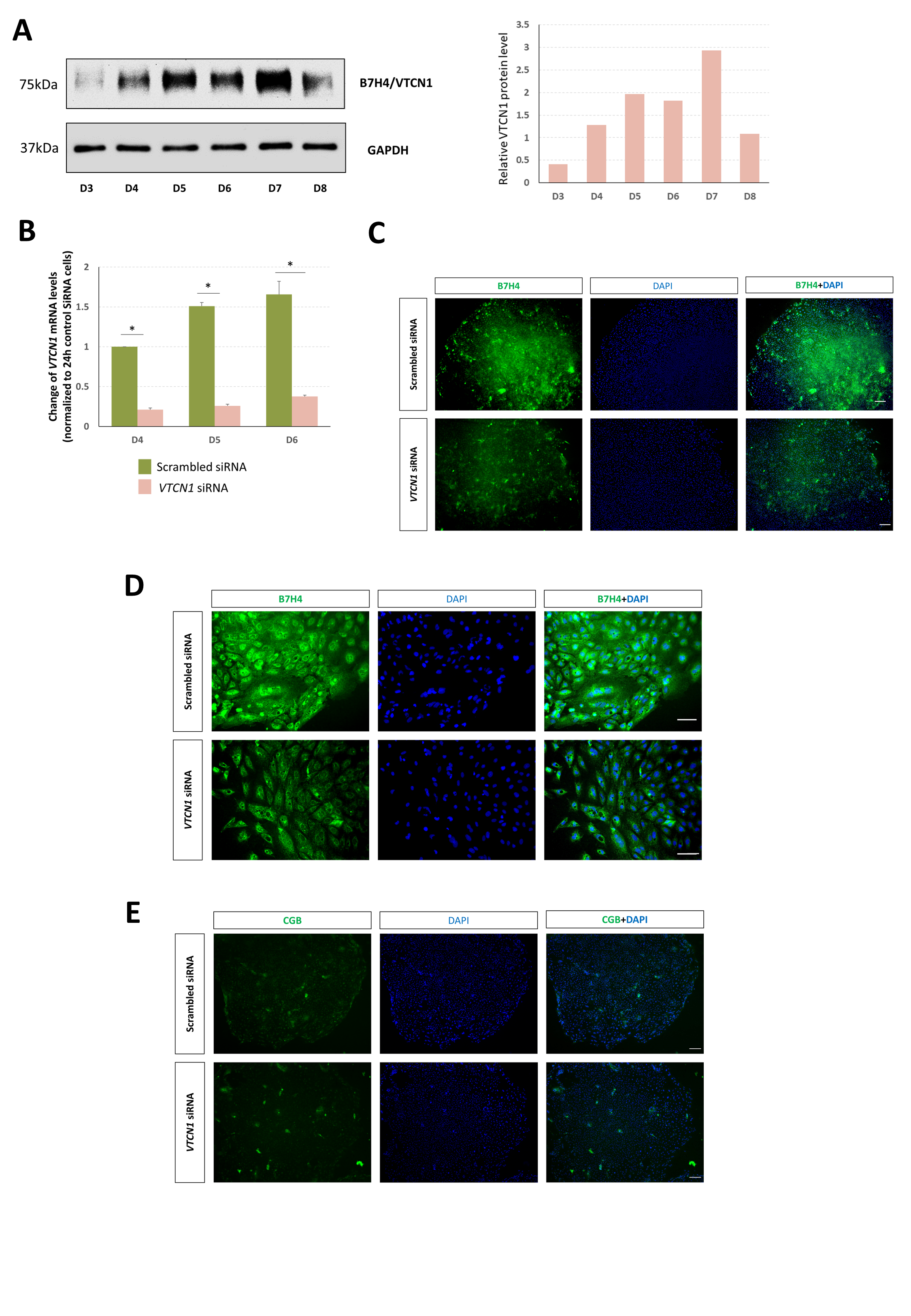

Supplement: Supplementary Figure 1 — (A) Time course for protein expression of B7H4 in BAP treated H1 hESCs from BAP d3 to d8. (B) BAP treated H1 hESCs were transiently transfected with scramble siRNA (control) or VTCN1 siRNA (VTCN1 KD) and cultured for up to 72 h. Assessments were made 24 h, 48 h and 72 h after transfection. Relative concentrations of VTCN1 transcripts were assessed by real-time PCR (n=3; i.e., three RNA preparations from three independent experiments). Data represents means ± SD, statistically significant values compared to scrambled control siRNA-exposed cells are indicated by asterisks (p<0.05). (C–E) BAP treated H1 hESCs were transiently transfected with scramble siRNA (control) or VTCN1 siRNA (VTCN1 KD) and kept in culture for 3 d after transfection. B7H4 (C, D) and CGB (E) protein expression was assessed by immunofluorescence microscopy (Scale bar, 100 µm). [file Image_1.tif]

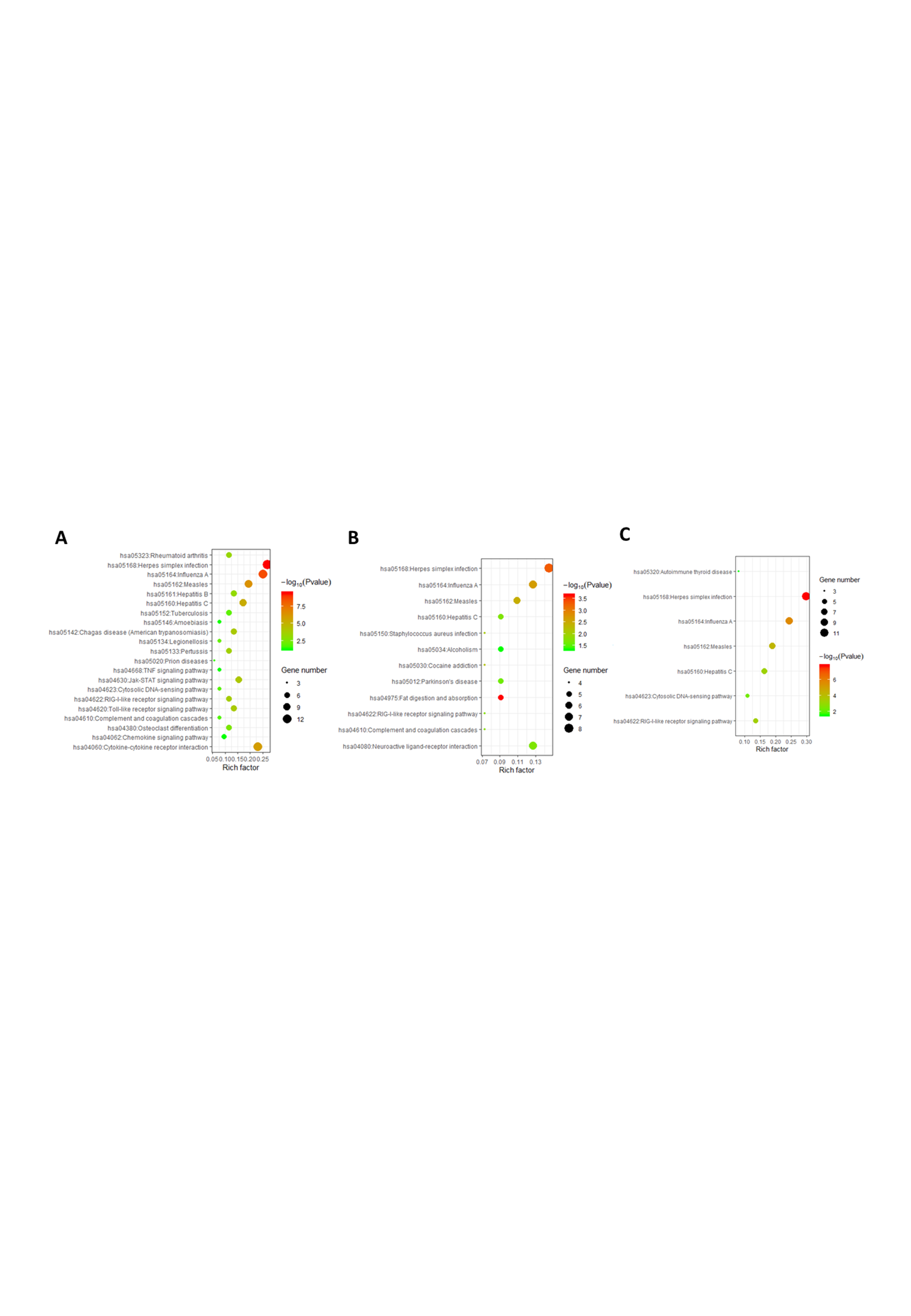

Supplement: Supplementary Figure 2 — KEGG pathway analysis of differentially expressed genes after exposure to VTCN1 siRNA and a matched scrambled siRNA control. (A) Genes upregulated 24 h after transfection, (B) genes upregulated 48 h after transfection, (C) genes upregulated 72 h after transfection. [file Image_2.tif]

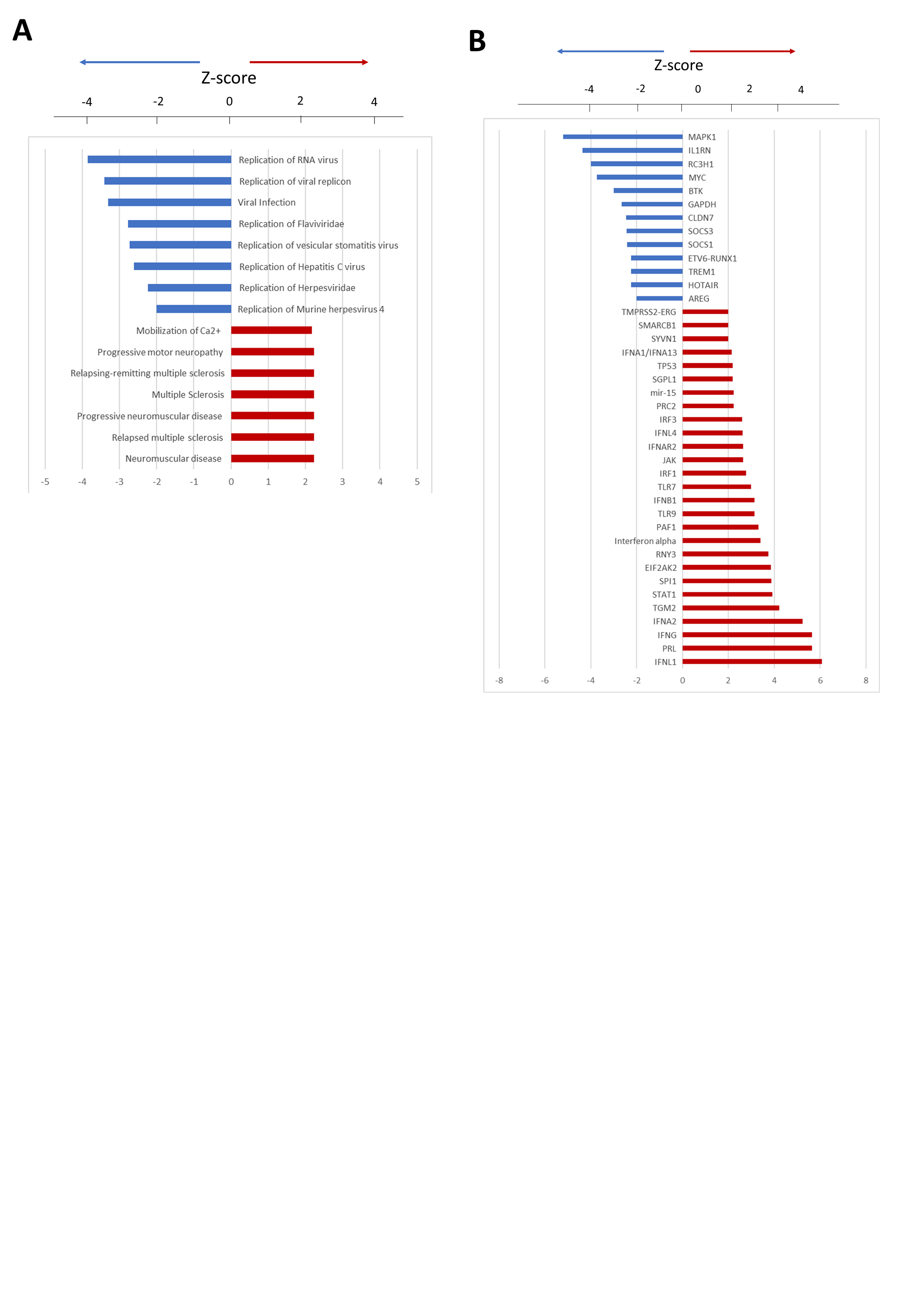

Supplement: Supplementary Figure 3 — Assessment of gene activation patterns for differentially expressed genes after exposure to VTCN1 siRNA and a matched scrambled siRNA control. (A) Affected functional categories 48 h after transfection. (B) Activated genes 48 h after transfection. [file Image_3.tif]

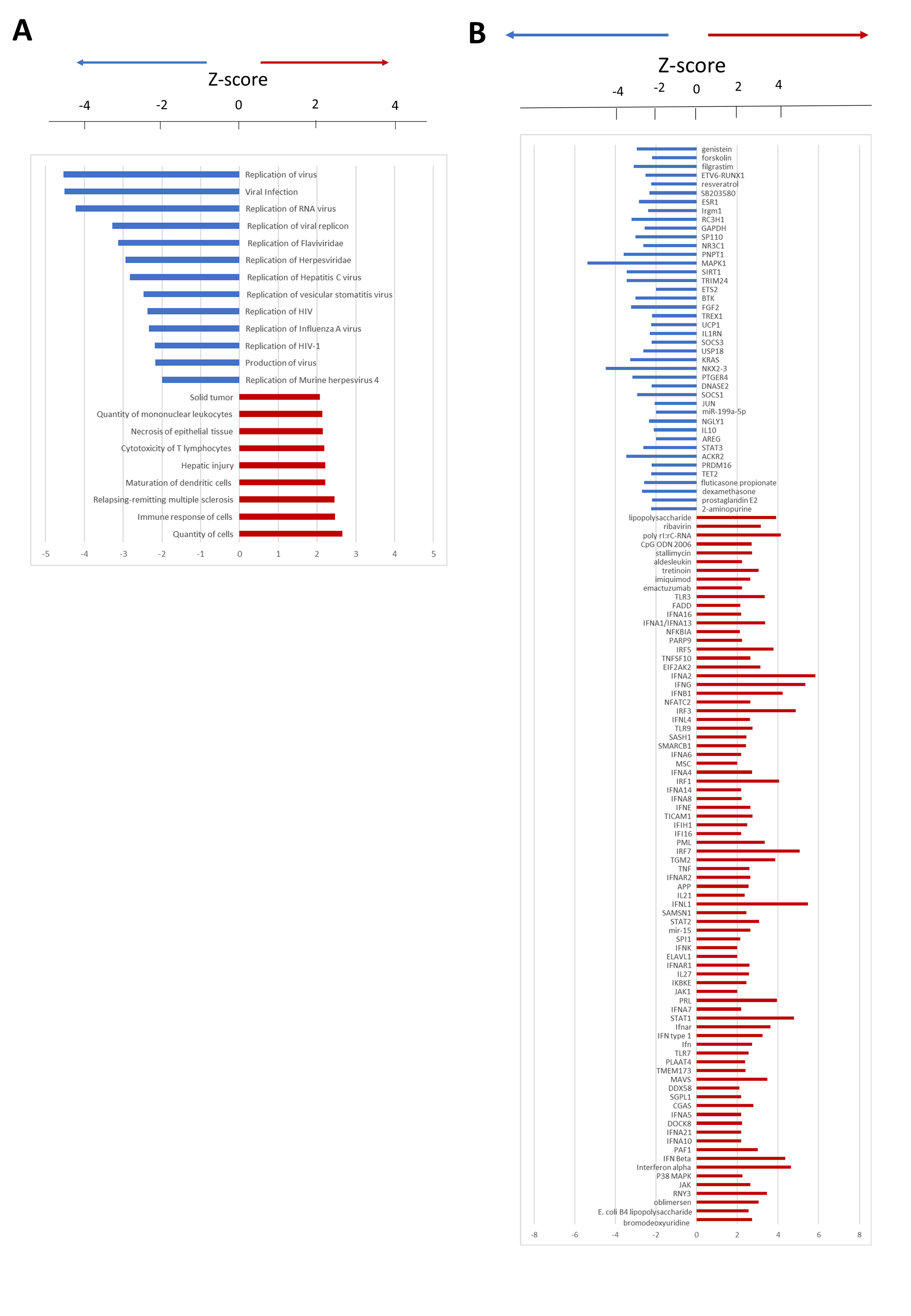

Supplement: Supplementary Figure 4 — Assessment of gene activation patterns for differentially expressed genes after exposure to VTCN1 siRNA and a matched scrambled siRNA control. (A) Affected functional categories 72 h after transfection. (B) Activated genes 72 h after transfection. [file Image_4.tif]

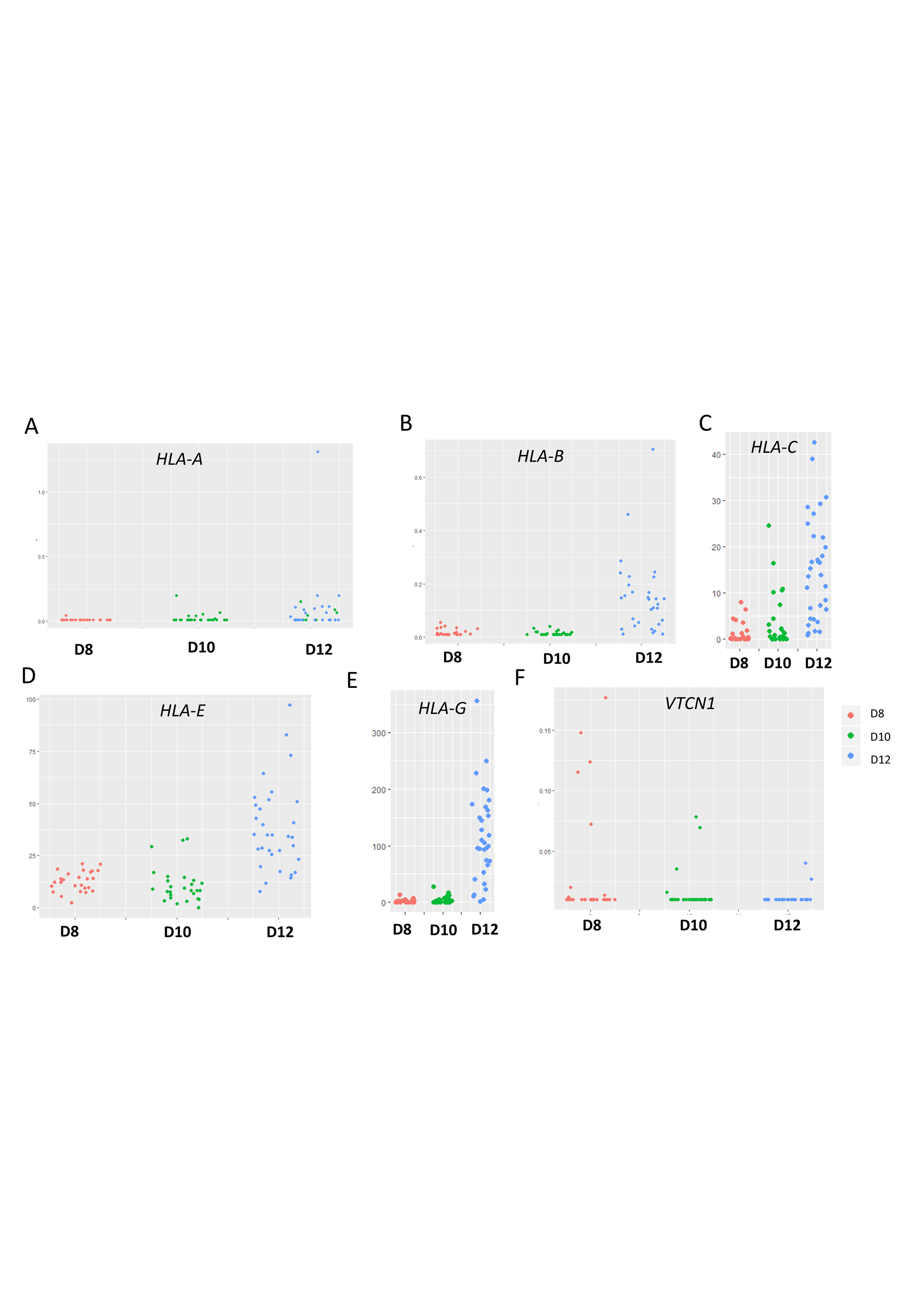

Supplement: Supplementary Figure 5 — Data were generated in a model of human extended blastocyst culture (26, 83) and were analyzed by using the publicly-available databases generated from that work. (A-F) MHC-I expression in early pregnancy. FPKM values of the MHC-I genes: HLA-A (A), HLA-B (B), HLA-C (C), HLA-E (D), HLA-G (E) and of VTCN1 (F) in small cells of human embryos on d8, d10 and d12. The authors suggest these small cells represent mononucleated CTB. [file Image_5.tif]
